# Trace Metal Content of Sediments Close to Mine Sites in the Andean Region

**DOI:** 10.1100/2012/732519

**Published:** 2012-04-19

**Authors:** Cristina Yacoub, Agustí Pérez-Foguet, Nuria Miralles

**Affiliations:** ^1^Grup de Recerca en Cooperació i Desenvolupament Humà (GRECDH), Departament d'Enginyeria Química, ETSEIB, Universitat Politècnica de Catalunya, Avenida Diagonal 647, 08028 Barcelona, Spain; ^2^GRECDH-LaCÁN, Departament de Matemàtica Aplicada 3, ETSECCPB, Universitat Politècnica de Catalunya, Jordi Girona 1-3, 08034 Barcelona, Spain; ^3^Departament d'Enginyeria Química, Universitat Politècnica de Catalunya, Avenida Diagonal 647, Edifici H Planta 4a, 08028 Barcelona, Spain

## Abstract

This study is a preliminary examination of heavy metal pollution in sediments close to two mine sites in the upper part of the Jequetepeque River Basin, Peru. Sediment concentrations of Al, As, Cd, Cu, Cr, Fe, Hg, Ni, Pb, Sb, Sn, and Zn were analyzed. A comparative study of the trace metal content of sediments shows that the highest concentrations are found at the closest points to the mine sites in both cases. The sediment quality analysis was performed using the threshold effect level of the Canadian guidelines (TEL). The sediment samples analyzed show that potential ecological risk is caused frequently at both sites by As, Cd, Cu, Hg, Pb, and Zn. The long-term influence of sediment metals in the environment is also assessed by sequential extraction scheme analysis (SES). The availability of metals in sediments is assessed, and it is considered a significant threat to the environment for As, Cd, and Sb close to one mine site and Cr and Hg close to the other mine site. Statistical analysis of sediment samples provides a characterization of both subbasins, showing low concentrations of a specific set of metals and identifies the main characteristics of the different pollution sources. A tentative relationship between pollution sources and possible ecological risk is established.

## 1. Introduction

Mine water pollution is a widespread international problem and one of the most severe forms of aquatic pollution. Specifically, acid mine drainage is one of the main causes of water pollution worldwide and has emerged as a major environmental problem over the last few decades [1–5]. Current and traditional mining activities have contaminated river channels and floodplains in many parts of the world with concentrations of metal-rich waste that may pose a risk to human livelihood and sustainable development [6]. Sediments in rivers polluted by acid mining drainage consist of a complex mixture of various geochemical fractions containing potentially toxic metals. The mobility of such hazardous metals is highly dependent on their specific chemical forms or different binding forms rather than on total element concentrations. Consequently, knowledge of metal partitioning between the different geochemical fractions is particularly useful for determining the bioavailable fraction and the risk of ecotoxicity [7–10].

South America has a long history of mining and many of the oldest mines are still active. Production in this sector has increased dramatically over the last fifteen years, accompanied by changes in public policy, many related social conflicts, shifts in patterns of natural resource and land ownership, and a rapid expansion of business-led social responsibility including the promotion of community development programs [11]. Due to the geomorphologic characteristics of the Andean region, there is a strong presence of trace metals in the environment. For this reason, mining activities are sometimes understood as just one more contributor to a total amount of trace metals. Despite of sediment relevance to assess mine pollution, sediment analyses are not usual in the region. This is the case of Jequetepeque River Basin (Peru), where sediment analyses have not been performed previous to the study presented here. In this context, the aim of this study is to contribute to the knowledge of availability of trace metals close to mine sites, and if it represents a possible toxic hazard or not. Due to some constraints, most notably economic resources, we were limited to only a few points of monitoring in spite of the intensive literature research of the site made before.

Sequential extraction schemes (SESs) are widely used for assessing trace metal mobilization and bioavailability by providing information on the distribution or partitioning of trace metals in soils and sediments. This information is particularly valuable in risk assessment and is not provided by sediment digestion procedures. SES assesses the availability, mobility, or persistence of trace metals to determine levels of retention and release (relative to readsorption and redistribution processes). In 1987, the Community Bureau of Reference (BCR) (now the Standards, Measurements and Testing Programme, SM&T) produced a standard protocol for the extraction of metals from soils and sediments to solve the problems caused by the wide variety of SES in existence at that time and the lack of comparability of results [3, 12–15]. The protocol uses fixed operation conditions (i.e., extractant agents, time and form of shaking, operation sequence, etc.), which ensures that results obtained in different laboratories can be compared accurately [16]. Therefore, BCR-SES protocol is normally used to study heavy metal distribution in sediments [14, 17–22].

Following this protocol, study areas and monitoring points were selected by a previous revision of the area of interest. Metal concentrations in sediment samples were performed by taking nine points located close to mine sites in Cajamarca, Peru. For this purpose, a specific monitoring campaign was designed based on the guidelines provided by the United States Environmental Protection Agency [23–29]. BCR-SES was used to identify chemically active forms in the sediment samples [20]. The sediment concentrations of Al, As, Cd, Cu, Cr, Fe, Hg, Ni, Pb, Sb, Sn, and Zn were analyzed for each fraction of the SES analysis. BCR-SES analysis for these metals and metalloids was performed following considerations from the literature [3, 15]. The sediment concentrations were compared with the concentrations established in the Canadian Sediment Quality Guidelines for the Protection of Aquatic Life [30], and a potential ecological risk was determined. A comparative study of metal concentrations and the corresponding ecological risks was made based on sediments collected from the mainstream and tributaries of two subbasins [31]. The sediment analysis data was processed using principal component analysis (PCA) and hierarchical cluster analysis (HCA), which identify similarities and differences between the data, to obtain more information about the pollution sources.

## 2. Methodology

### 2.1. Study Area

**Figure 1:**
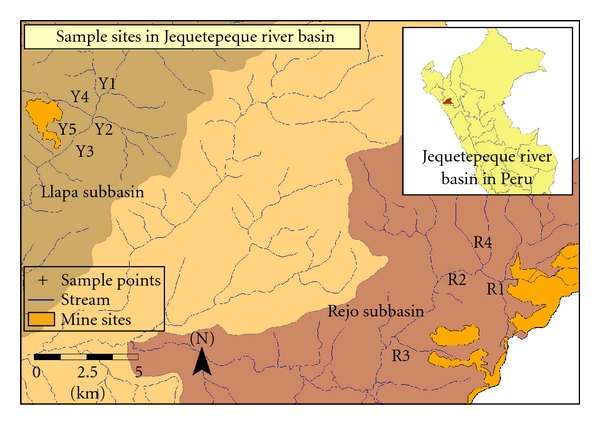
Map of monitoring sites. Y1, Y2, Y3, Y4, and Y5 in Llapa subbasin and R1, R2, R3, and R4 in Rejo subbasin in the Jequetepeque River Basin, Cajamarca, Peru.

The Jequetepeque watershed (4372.5 km^2^) is located in northern Peru (Figure 1). The river flows east to west from the Andes to the Pacific Ocean. Annual average precipitation on watershed ranges from 0 to 1100 mm and its altitude varies from 0 to 4188 meters above sea level (m.a.s.l.). The Gallito Ciego reservoir (400 m.a.s.l.) separates the upper-middle part of the watershed from the lower part and stores water used to supply the population of large coastal cities and for extensive agriculture. The upper-middle part of the watershed covers an area of 3564.8 km^2^. Approximately 80% of the population is rural. The main activities are agriculture, livestock, and mining, and there are no other productive activities such as tanning or intensive farming [32]. Two study areas were selected, each one in a different subbasin: Llapa and Rejo (see Figure 1). The Llapa subbasin includes the Sipán SL mine and the Rejo subbasin includes part of the Minera Yanacocha SRL mine. In both mines, gold extraction has been performed with cyanide leaching and the Sipán SL mine was closed in 2005. Thus, trace metal release could be caused by mining activities or could simply be due to the naturally high levels found in the Andes.

The study areas were selected on the basis of the following criteria: (i) USEPA Guidelines [25–29]; (ii) proximity to the main mining activities; (iii) hydrology, geology, geomorphology, erosion, and topography [32–35]; (iv) previous evidence of toxicology or spillage; (v) available data from studies of water quality [33, 34, 36]; (vi) feasibility of monitoring activities, particularly regarding physical access to sites and approval of local residents.

### 2.2. Sediment Samples

This survey was conducted in October 2008. Nine sampling points were established in the two study areas following above-mentioned criteria, 5 in the Llapa subbasin and 4 in the Rejo subbasin (see Figure 1). In the Llapa subbasin, points Y1, Y2, and Y3 were located in the mainstream and Y4 and Y5 in tributaries (which are assumed to come into contact with acid drainage). Y1 was upstream of the contact area with polluted water. In the Rejo subbasin, points R1, R2, and R3 were in the mainstream and R4 was in a tributary (without known contact with polluted waters). A total of 10 sediment samples were collected in plastic bottles using a core sampler. One sample was taken as a field replicate of sediments following the USEPA recommendations [28, 29]. Samples were taken from the nearest point on the river bank, were stored in plastic bottles, and kept cool in the field [28]. The sediment samples were surface sediments (0–5 cm of depth). Three sediment subsamples were collected and mixed at each site (within 100 m of the river) as a composite sampling procedure in order to obtain a representative sample [28]. The sub-samples were mixed in the field in a tray with a total volume of 1 L. The sediments were composed mostly of clay and silt, and the percentage of rocks varies depending on the sampling point, from 0.9% to 85%, with most cases around 6%. Electrical conductivity and pH were directly measured in the field. The sediment samples were analyzed at the Corrosion and Protection Institute Laboratory of the Pontifical Catholic University of Peru in Lima.

Sediment samples were dried at 60°C until constant weight [37] and sieved to obtain a particle size of <63 *μ*m (UNE Standard 7050), which retains trace metals [12–14, 37–41]. Dried samples were stored in polyethylene bottles.

### 2.3. Sequential Extraction Scheme and Sediment Analysis

The BCR procedure, together with its related phases and its respective reagents, is summarized below and full details can be found elsewhere [15, 16, 20].

*Step  1* consists in extracting exchangeable water- and acid-soluble species, which are weakly bound metals retained on the sediment surface by relatively weak electrostatic interactions and can be released by changes in ionic competition or affected by small pH changes. For each sample, 40 mL of 0.11 mol dm^−3^ acetic acid was added to 1 g of sediment in a 100 mL centrifuge tube and shaken for 16 h at room temperature. The extract was separated from the solid residue by centrifugation and decantation of the supernatant liquid into a high density, polyethylene container. The container was stopped and the extract was stored at 4°C. The residue was washed by adding 20 mL of water, shaking for 15 min, and finally centrifuging the resulting suspension.

*Step  2* consists in extracting reducible species contained in iron and manganese oxides, which are released due to their instability under reducing conditions. In this step, 40 mL of 0.1 mol dm^−3^ hydroxylamine chloride (adjusted to pH 2 with nitric acid) was added to the residue from Step  1 in the centrifuge tube, and the extraction and wash were performed as described above.

*Step  3* consists in extracting oxidizable species, which are trace elements bound to various forms of organic matter. The degradation of organic matter under oxidizing conditions is responsible for releasing trace elements. 10 mL of solution 8.8 mol dm^−3^ H_2_O_2_ (adjusted to pH 2 with nitric acid) was added carefully, in small aliquots to avoid losses due to violent reaction, to the residue from Step  2 in the centrifuge tube. The sample was digested at room temperature for 1 h with occasional manual shaking. After that, we used a water coating (about 85°C) until evaporation. When the samples were dry, the entire process was repeated. Next we added 50 mL of ammonium acetate 1 mol dm^−3^, and it was shaken for 16 h at room temperature. Again, the extraction was performed as described in Step  1.

*Step  4* consists in extracting residual species, which are constituent elements of the lattice mineral structure that are not easily released into the water. 5 mL of HCl and 15 mL of HNO_3_ (both trace analysis quality) were added in a Pyrex container in a sand coating for 3 h at 150°C. Finally, the sample was centrifuged and the supernatant was separated in a polyethylene tube and kept at 4°C until the analyses were performed.

The consideration of the metal and metalloid content in sample sediments was obtained as the sum of each step, taking into account that no significant differences between total metal content by aqua regia digestions and the sum of the extracted metals by BCR procedures [12]. Assessment of trace metal loads released from mining was carried out by analyzing the ratios between the average mainstream and tributary values of metal content in sediments. In order to evaluate the associated risk of the metal loads, the available metal fraction was calculated as the sum of the three first fractions of SES [13, 14, 42].

Metal contents were determined by atomic absorption spectrometry by hydride generation based on SM 3114 B-C-2005 for As, cold vapor atomic absorption spectrometry based on EPA 245-1 method for Hg, and inductively coupled plasma optical emission spectrometry (ICP-OES model OPTIMA 3000DV-Perkin Elme) based on EPA 200.7 for the other metals. The detection limits were 0.1 mg/kg for Cd, Cu, Cr, and Pb, 0.05 mg/kg for As, and 0.02 mg/kg for Hg. All used reagents were of analytical grade. The efficiency of the analysis was assessed by three replicates. The relative standard deviations obtained varied from 5 to 11% and are considered satisfactory given the complex nature and the differences between the samples of the sediment matrix.

### 2.4. Statistical Analysis

Multivariate statistical analysis was applied to the sediment data looking for possible characterization of different pollution sources. Data was processed using PCA and HCA [13, 14, 43] by SPSS v15.0 software package.

First, PCA was applied to the 9 samples, characterized by 13 metal and metalloid concentrations as the sum of the values obtained in each step of the SES. The principal component and eigenvectors of Pearson correlation matrix values were computed. Next, rotation of principal components was carried out using the Varimax normalized algorithm, which facilitates interpretation of the principal component by maximizing the variance of the extracted factors and reducing the uncertainties of initial unrotated factor loading [14]. Varimax rotation was applied to the principal components that contribute more than 5% of the total variance of the data set. Finally, HCA was applied to the 9 samples, expressed in terms of the four rotated factors. HCA is the most common cluster analysis method in environmental analysis and group samples according to data similarities. The most similar points are grouped in a cluster, and the process is repeated until all points belong to one cluster [38]. Statistical calculations were performed with SPSS v15.0 software package, and decimal logarithm transformation was used to improve the statistical analysis. Kurtosis and skewness statistics indicate that the transformed variables are closer to a normal distribution than the original variables for almost all metal concentrations. A threshold value of 0.001 mg/kg was set for Hg and Sb concentrations for results below concentration detection limits.

## 3. Results and Discussion

### 3.1. Sediment Metal Content

Different trends were observed between both subbasins. First, the concentration range, median, mean, standard deviation (SD), and skew of trace metal in sediment samples from the mainstream (Y1, Y2, Y3) and tributaries (Y4, Y5) of the Llapa subbasin are presented in Table 1. In this area, tributary sampling points were located in streams flowing directly from the mine site (Figure 1).  Table 2 shows the same type of data as Table 1 but for the mainstream (R1, R2, and R3) and tributary (R4) monitoring points in the Rejo subbasin. In this case, R4 was located in a stream that does not come into contact with mine drainage and could therefore be considered nonpolluted.

In the Llapa subbasin (Table 1), concentrations of Cr, Ni, and Sn did not differ significantly between samples, but differences were observed in the values of Al, Fe, and Zn. These findings are in agreement with the literature [44, 45]. Slightly high values were found in Y2, located at the mainstream, and in Y4 and Y5, located at tributaries. Mainstream and the tributary point Y5 concentrations showed comparatively greater differences of the remaining elements (As, Cd, Cu, Pb, and Sb). As was more than sixteen times higher in tributaries than in the mainstream, and Cu, for which a ratio of over 8 : 1 was obtained. In Rejo subbasin, concentrations of Al, As, Cr, Fe, Sb, and Sn were similar at all sampling points and small differences were found for Hg, Ni, and Pb. In contrast, considerable differences were found for Cu, Cd, and Zn showing comparatively higher concentrations in R1. Lowest metal concentrations of the whole data set for almost all of the metals were obtained in the R4 tributary.

Taking into consideration that high pollutant concentrations accompanied by high standard deviations suggest anthropogenic sources, pollution due to mining is confirmed for both subbasins. Additionally, metal concentrations from monitoring points near mine sites exceed the levels present in unpolluted areas, such as the tributaries or mainstream. Homogeneous distribution across the site (R4 in Rejo subbasin and Y1, Y2, and Y3 in Llapa subbasin) confirm this concern especially in Llapa subbasin with lower standard deviations. Therefore, mine pollution is present in Llapa subbasin for As, Cd, Cu, Pb, and Sb and in Rejo subbasin for Cu, Cd and Zn.

### 3.2. Ecological Risk Assessment

**Figure 2:**
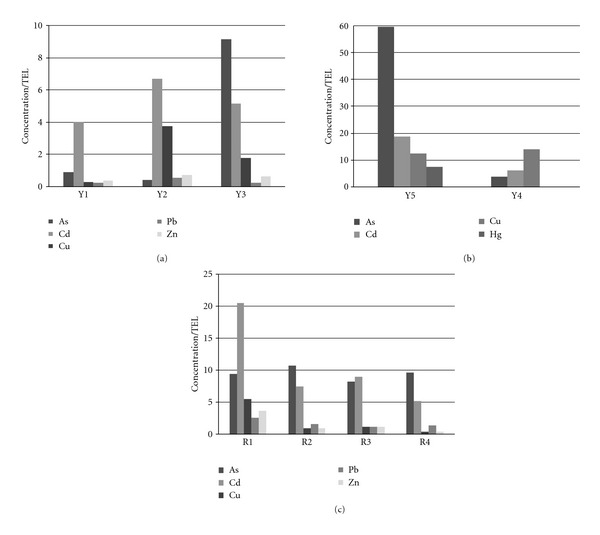
Ratios of trace metals with threshold effect level TEL in sediments from the mainstream (Y1, Y2, Y3) and tributaries (Y4 and Y5) of the Llapa subbasin, and from the mainstream (R1, R2, R3) and tributary (R4) of the Rejo subbasin.

The potential toxicological effect of sediment trace metal content was assessed in this section. For this, the sediment concentrations were compared with the concentrations established in the Canadian Sediment Quality Guidelines for the Protection of Aquatic Life [30]. Two levels of ecological risk are considered for sediment samples: the threshold effect level (TEL) below which adverse biological effects are expected to occur rarely, and the probable effect level (PEL) above which adverse effects are expected to occur frequently. The data is summarized in Figure 2. Table 1 shows that concentrations of Cd and Cu in the Llapa subbasin were above the PEL in all tributary samples, and concentrations of Pb and Zn were above the TEL in 33% of the tributary samples. In addition, Hg concentrations were above the PEL in approximately 33% of both mainstream and tributary samples. As gives values far above PEL for Y5 and downstream from this tributary (Y3) values were also above PEL.

Table 2 shows that concentrations of As in the Rejo subbasin were above the PEL in all samples (mainstream and tributary), while Cd is above PEL only in mainstream samples. This is congruent with other findings present in the literature [44]. Concentrations of Cu, Pb, and Zn were above the PEL in 33% of the mainstream samples.

In conclusion, according to Canadian guidelines, there is a risk of toxicity to aquatic life by As, Cd, Cu, Hg, Pb, and Zn in the studied area. This is particularly frequent for As, Cd, and Cu in the Llapa subbasin, whereas considerable toxicological effects caused by Pb and Zn are expected in Rejo subbasin. These results are consistent with the total content of trace metals in sediments nearby mine sites, suggesting that mining not only cause pollution, but also affects the aquatic life.

### 3.3. Sequential Extraction Scheme Analysis

**Figure 3:**
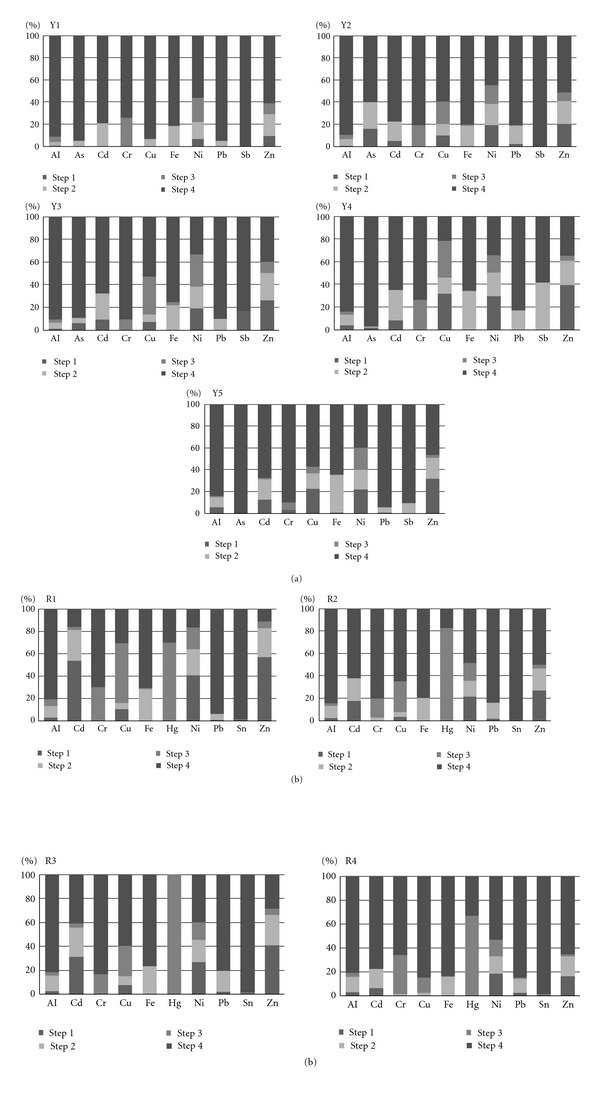
(a) Partitioning of speciation of trace metals in sediments from the mainstream (Y1, Y2, Y3) and tributaries (Y4 and Y5) in the Llapa subbasin. (b) Partitioning of speciation of trace metals in sediments from the mainstream (R1, R2, R3) and tributary (R4) in the Rejo subbasin.

The potential environmental risk posed by heavy metal content in sediments is dependent on both total content and speciation. The concentrations of metals in all monitoring points in each sequential extraction step (acid-extractable, reducible, oxidizable, and residual) are presented in Table 3 and Figure 3. This figure shows the speciation and partitioning of trace metals in sediments from the mainstream and tributaries of the Llapa (Figure 3(a)) and Rejo subbasins (Figure 3(b)). It should be noted that almost the entire content sum of Sn and Sb is present in the residual fraction in all samples of Llapa and Rejo subbasin, respectively.

Step  1 is considered an indicator of metal reservoir or pollution potential and also provides information of more recent contamination [46]. The results show that Zn is the most important in this fraction due to its high percentage value in samples R1, R3, Y4, and Y5 (40%). Similar trends were found for previous work in regions with this type of pollution [13, 14, 31]. Additionally, high contents of Ni, Cu, and Cd are also relevant (above 20%) increasing the potential pollution risk. Therefore, contamination by Zn, Ni, Cu, and Cd could be attributed to mining in its immediate area.

The reducible fraction (Step  2) is related to metals bound to Mn and Fe oxyhydroxides, which are released due to their instability under reducing conditions. Previous studies found significant associations between trace metals and Fe and Mn oxides in sediments of regions that receive discharges of industrial effluents [45]. Fe, Zn, Cd, and Ni account 20% of the total content in this fraction for all the samples. Zn and Cd are primarily due to anthropogenic sources according to sediment metal content analysis and Step  1. These findings are consistent with contaminated sediments and soils in the literature [7, 8, 42]. The presence in this step could be as a result of continuous input of these metals from mining. Concerning Fe and Ni distribution, the highest contents were found at sample points close to mine sites, indicating that this may be attributed to lithogenic and anthropogenic activities. These findings are in agreement with the literature [14, 45, 47]. Approximately 20% of As is bounded to Fe in Y2 probably adsorbed onto Fe oxy-hydroxides [7]. Approximately 40% of the total concentration of Fe and Sb is present in the second fraction, for the specific case of point Y4, and Y5 in a lesser extent, similar to findings from a contaminated zone due to a mine spill [8]. Downstream from these points, approximately 20% of Sb is found in the first fraction. Sb is mainly detected in the residual fraction or bound to oxy-hydroxides due to its low solubility; therefore it is surprising to find a considerable amount of Sb in Step  1. However, low concentrations of Sb are present in this sample, meaning that it does not present an environmental risk.

Trace metals associated with organic material are bound to the oxidizable fraction (Step  3) and may be mobilized by decomposition processes. Metals bound to sulfides might be extracted in this fraction. Considerable percentage of Hg is related to this fraction (23%) for all samples from Rejo subbasin, being a significant threat to the environment due to the high toxicity of Hg. Cr and Cu are also present in this fraction. It has to be noted that Hg and Cr are found in low and homogeneous concentrations in all samples; therefore lithogenic origins can be related to this fraction. The presence of Cu in the third fraction is only related to Llapa subbasin and R1. Similar to Fe and Ni distribution in Step  2, the presence of Cu in all the Llapa samples could be related to lithogenic origins, while in R1 could be primarily related to anthropogenic inputs. Differences between samples for Cu in this fraction are in agreement with previous work [7, 31, 47].

In summary, the order of relative abundance within the mobile fraction in all the samples (considering the percentage of metals extracted as the sum of the three first fractions) was Zn (69%) > Ni (61%) > Cu (58%) > Cd (47%) > Fe (26%) > Hg (23%) > Cr (21%) > Al (15%) > Sb (12%) = Pb (12%) > As (2%) = Sn (2%). Elevated concentrations of metals in residual fractions (low percentage of mobile fraction) indicate that the sediments are relatively unpolluted, then lithogenic origins can be related to Al, Sb, Pb, As, and Sn [45].

Summarizing, Zn, Ni, Cu, and Cd showed the greatest amounts of trace metals in the bioavailable fractions being a significant threat for the environment. The risk is particularly higher for Rejo than Llapa subbasin where high and available concentrations of Cd, Cu, and Hg—and to a lesser extent Pb and Zn—were found in sediment samples close to mine sites (Tables 1 and 2). The Llapa study area illustrates different behaviors at specific points for As, Fe and Sb taking into account the main results outlined above (Figure 3). However, content of As, Fe, and Sb in these related samples are low (Table 1), therefore their presence in movable fractions do not represent an environmental risk. Despite low values given for Cr and Hg, their availability has to be considered also as a potential risk effect on the environment due to its their specific toxicity, so subsequent studies must be carried out in order to evaluate their environmental risk, especially in Rejo subbasin.

### 3.4. Statistical Analysis

Table 4 shows loadings of metals on the principal components with the cumulative percentages in each case. The four eigenvalues greater than one were taken into account in further analysis, the others were discarded. These four factors/components accounted for approximately 90% of the total variance. The corresponding rotated factors were obtained by Kaiser's varimax rotation. All four contributed to a similar extent to the overall variance (from 26.5% for the first factor to 20% for the fourth). Table 4 shows the factor scores in terms of 13 metal concentrations for all sediment samples. Communalities (*h* ^2^, square of loading) are included; all values are over 0.8, which confirms the decision to use four factors. Factor 1 is heavily dominated by Mn and Zn and by Cd, Ni, Pb, and Cu to a lesser extent. Cr has a negative influence on this first factor, so low values of Cr correlate with high values of Factor 1. Factor 2 is highly correlated to As and Fe, and to a lesser extent on Cd, Cu, and Ni. Factor 3 is dominated by Sn and Hg, and it also depends on Pb and Zn. Factor 4 depends heavily on Al and Sb, less strongly on Cu, and weakly on Ni. Cr also has a negative influence on Factor 4.

**Figure 4:**
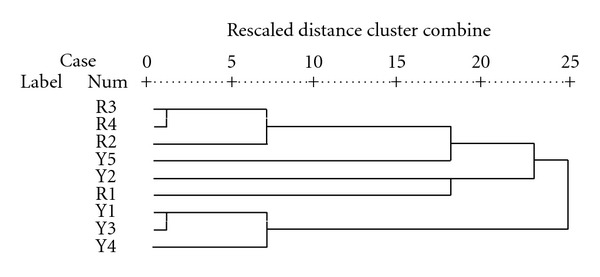
Dendogram representing the results of the HCA analysis of sediment samples.

HCA was applied to the nine samples, expressed in terms of the four factors. The dendogram is shown in Figure 4. The largest jump in rescaled distance cluster is found from the values 7 to 18, see Figure 4. The members of the five clusters are as follows: Cluster 1 is formed by R2, R4, and R3; Cluster 2 by Y5; Cluster 3 by Y2; Cluster 4 by R1; Cluster 5 by Y1, Y3, and Y4. Note that Cluster 1 only contains sampling points from the Rejo subbasin and Cluster 5 only contains points from Llapa. The results for each sample are expressed in terms of rotated factors in Table 5.

The samples are grouped according to HCA clusters, which help to identify the main characteristics of each one. Clusters 2, 3, and 4 show the highest dependence on the 4 factor loadings (with values of around 2 depending on each cluster), while Clusters 1 and 5 show lower values (less than −1) for all factor loadings. This means that the first set of clusters is characterized by high values of certain metals (each cluster is related to different factors), whereas the second set of clusters is characterized by low values of almost all trace metals. This interpretation of the HCA results suggests that Cluster 1 may represent a characteristic *natural *profile of metal content in the Rejo subbasin (with relatively low values for most metals, particularly those in Factor 4, with the exception of those in Factor 3), and Cluster 5 a characteristic *natural *profile of the Llapa subbasin (with relatively low values for most metals, particularly those in Factor 3, with the exception of those in Factor 4). In contrast, the configuration of Clusters 2, 3, and 4 could be related to different sources of pollution in the subbasins (leading to a different relative importance of Factors 1, 3, and 4). Rejo subbasin could be characterized by relatively low natural concentrations of Al, Sb, Cu, and Ni, and the Llapa subbasin by relatively low natural concentrations of Hg, Pb, and Cr, which appears to be consistent with the results in Tables 4 and 5.

Pollution can be expected in the mainstream of the Rejo subbasin and will increase in areas closer to the source of mine drainage, leading to high concentrations of Cd, Cu, Ni, Pb, and Zn. Two pollution profiles are found in the Llapa subbasin: one in a tributary at the sampling point closest to the mine, where very high concentrations of As, Cd, Cu, Fe, Ni, and Sb were recorded, and the other in the mainstream located downstream of the tributaries carrying mine drainage, where high concentrations of Al, Cu, Cr, Hg, Ni, Pb, and Sn were recorded. Cu and Pb are also found in the polluted areas of Rejo subbasin though they do not form part of its expected natural profile. The PCA analysis produces similar results to the previous assessments: low values are recorded at point Y1 in the Llapa subbasin, which is in the mainstream located upstream of the contact with mine drainage, and at point R4 in the Rejo subbasin.

Finally, as explained above, in the Llapa subbasin the Hg toxicity threshold is exceeded frequently, although relatively low values can be expected if we consider the hypothetical natural profile. In the Rejo subbasin, the Cu toxicity threshold is very likely to be exceeded (in the mainstream), although relatively low values are expected taking into account the hypothetical natural profile. Consequently, the putative link between toxic risk and pollution hotspots is confirmed, at least in the studied area.

## 4. Conclusions

The following conclusions can be drawn from this study.

Sediment samples from the Llapa subbasin showed considerable differences in the concentrations of As, Sb, Cd, Cu, Pb, and Hg between the mainstream and the tributary point Y5 (the closest to the mine). In the Rejo subbasin, considerable differences in the concentrations of Cd, Cu, and Zn were found between mainstream (downstream of flow from the mine site) and tributary samples.

Exposure at aquatic life to toxicity in the studied area is frequently caused by sediment contamination with As, Cd, Cu, Hg, Pb, and Zn. As, Cd, and Cu concentrations are particularly high in the Llapa subbasin, and Pb and Zn concentrations in the Rejo subbasin.

Mobility of the metals, assessed by SES analysis, was Zn (69%) > Ni (61%) > Cu (58%) > Cd (47%) > Fe (26%) > Hg (23%) > Cr (21%) > Al (15%) > Sb (12%) = Pb (12%) > As (2%) = Sn (2%). Therefore, Zn, Ni, Cu, and Cd could be considered as a significant threat for the environment in all the monitoring points. In addition, relevant percentages of Cr and Hg are available in some points of Rejo subbasin and As, and Sb in Llapa. The risk is particularly high in the Rejo compared with the Llapa subbasin, where high and available concentrations of Cd, Cu, and Hg—and to a lesser extent Pb and Zn—were found.

Principal component and cluster analyses provide a plausible characterization of the natural (nonpolluted) metal content profiles of the watershed. Besides, sets of metals characteristic of different pollution sources are identified in the two subbasins: (i) pollution can be expected in the mainstream of the Rejo subbasin, particularly at the closest point to the mine drainage, where high concentrations of Zn, Cr, Ni, Cd, Cu, and Pb were found; (ii) two pollution profiles were identified in the Llapa subbasin: one in a tributary at the closest sampling point to the mine, which shows very high concentrations of As, Cd, Fe, Ni, and Cu,s and the other in the mainstream located downstream of the tributaries affected by mine drainage, where high concentrations of Al, Cu, Cr, Hg, Ni, Pb, Sb, and Sn were recorded.

The results of this study are the first step towards a comprehensive evaluation of metal discharges in Jequetepeque River Basin by mining activities. In this sense, their threat to the environment was assessed showing significant impacts in the studied areas.

## Figures and Tables

**Table 1:** Metal concentration (mg/kg), in sediment samples with grain size < 63 *μ*m for mainstream of Llapa Subbasin with median, mean, standard deviation, and skew for Y1, Y2, and Y3 (mainstream points); threshold effect level (TEL), and probable below level (PEL).

	Y1	Y2	Y3	Y4	Y5	Median^a^	Mean^a^	SD^a^	Skew^a^	TEL	PEL
pH	7.30	7.60	7.68	—^c^	3.44	7.60	7.53	0.2	−1.4	—	—
EC^b^	31.00	35.00	38.00	734.00	212.00	35.00	34.67	3.5	−0.4	—	—
AI	13687.3	19378.5	12649	24522.7	15265.5	13687.30	15238.27	3622.9	1.6	—	—
As	5.28	2.5	53.88	22.65	350.73	5.28	20.55	28.9	1.7	5.9	17
Cd	2.4	4	3.1	3.7	11.3	3.10	3.17	0.8	0.4	0.6	3.5
Cr	5.4	6.8	4.3	5.7	5.5	5.40	5.50	1.3	0.4	37.3	90
Cu	10.3	133.1	63.4	502	446	63.40	68.93	61.6	0.4	35.7	197
Fe	12397.4	13930	14478	17810.4	24884.25	13930.00	13601.80	1078.4	−1.2	—	—
Hg	n.d.^d^	1.02	n.d.	n.d.	1.26	0.00	0.34	0.6	1.7	0.17	0.49
Mn	749.3	1205.8	957.7	933.7	372.75	957.70	970.93	228.5	0.3	—	—
Ni	5.9	9.4	8.4	10.5	11.6	8.40	7.90	1.8	−1.2	—	—
Pb	8.1	20	8.9	9.5	35	8.90	12.33	6.7	1.7	35	91
Sb	0.9	1.2	1.2	1.2	4.75	1.20	1.10	0.2	−1.7	—	—
Sn	64.7	93.3	71.1	65.5	85.4	71.10	76.37	15.0	1.4	—	—
Zn	45	90.6	76	126.1	114.65	76.00	70.53	23.3	−1.0	123	315

^a^Calculated for mainstream samples (Y1, Y2, Y3); ^b^Ohms*1000; ^c^no available; ^d^no detectable (n.d.).

**Table 2:** Metal concentration (mg/kg) in sediment samples with grain size < 63 *μ*m for tributaries of Rejo subbasin with median, mean, standard deviation and skew for R1, R2, and R3 (mainstream points), threshold effect level (TEL), and probable below level (PEL).

	R1	R2	R3	R4	Median^a^	Mean^a^	SD^a^	Skew^a^	TEL	PEL
pH	—^c^	—	—	—	—	—	—	—	—	—
EC^b^	37	—	32	—	34.5	34.5	—	—	—	—
AI	15605	13574.6	9601.9	14330.4	13575	12927	3053.5	−0.9	—	—
As	55.93	63.3	48.78	56.82	55.9	56	7.3	0.0	5.9	17
Cd	12.3	4.5	5.4	3.1	5.4	7.4	4.3	1.6	0.6	3.5
Cr	3.3	7.6	4.8	6.7	4.8	5.2	2.2	0.9	37.3	90
Cu	198	34.4	42.8	16.6	42.8	91.7	92.1	1.7	35.7	197
Fe	15360.1	16810.3	15261	14731.7	15360.1	15810	867.3	1.7	—	—
Hg	0.1	0.17	0.1	0.09	0.1	0.12	0.0	1.7	0.17	0.49
Mn	3711.8	878.6	932.2	349.9	932.2	1841	1620.5	1.7	—	—
Ni	12	11.8	7	6.4	11.8	10.3	2.8	−1.7	—	—
Pb	92.6	54.7	40.5	47.9	54.7	62.6	26.9	1.2	35	91
Sb	n.d.^d^	n.d.	n.d.	n.d.	—	—	—	—	—	—
Sn	76.3	79.7	62.8	87.5	76.3	72.9	8.9	−1.5	—	—
Zn	452.7	122.2	149.9	55.3	149.9	241.6	183.3	1.7	123	315

^a^Calculated for mainstream samples (R1, R2, R3); ^b^Ohms*1000; ^c^no available; ^d^no detectable (n.d.).

**Table 3:** Concentrations (mg/kg) of all the sediment samples in each fraction of the BCR sequential extraction.

Sample	BCR Step	Al	As	Cd	Cu	Cr	Fe	Hg	Ni	Pb	Zn
Y1	Step 1	61.9	<0.05	<0.1	<0.1	<0.1	9.2	<0.02	0.4	<0.1	4.3
	Step 2	473	0.28	0.5	0.7	<0.1	2212.5	<0.02	0.9	0.4	8.7
	Step 3	647.2	<0.05	<0.1	<0.1	1.4	120.6	<0.02	1.3	<0.1	4.6
	Step 4	12505.2	5	1.9	9.6	4	10055.1	<0.02	3.3	7.7	27.4

Y2	Step 1	119.7	0.4	0.2	13.6	<0.1	50.6	<0.02	1.8	0.5	18.6
	Step 2	1154.7	0.6	0.7	13.4	<0.1	2564.7	<0.02	1.8	3.2	18.8
	Step 3	716.2	<0.05	<0.1	27.0	1.3	167.5	0.02	1.6	0.1	6.6
	Step 4	17387.9	1.5	3.1	79.1	5.5	11147.2	1.02	4.2	16.2	46.6

Y3	Step 1	153.1	3.29	0.3	4.6	<0.1	24.3	<0.02	1.6	<0.1	20
	Step 2	675.9	2.29	0.7	4	<0.1	3136.9	<0.02	1.6	0.9	18.1
	Step 3	381.2	0.3	<0.1	21.3	0.4	407.5	<0.02	2.4	<0.1	7.7
	Step 4	11438.8	48	2.1	33.5	3.9	10909.3	<0.02	2.8	8	30.2

Y4	Step 1	910.6	0.35	0.3	159.8	<0.1	26	<0.02	3.1	<0.1	49.6
	Step 2	2356.1	0.3	1	70.9	<0.1	6014	<0.02	2.2	1.6	26.8
	Step 3	623.6	<0.05	<0.1	161.4	1.5	108.5	<0.02	1.6	<0.1	5.9
	Step 4	20632.4	22	2.4	109.9	4.2	11661.9	<0.02	3.6	7.9	43.8

Y5	Step 1	815.3	0.3	1.5	101.2	0.2	194.5	<0.02	2.55	0.2	36.8
	Step 2	1482.3	0.35	2.1	63.7	0.1	8542.5	<0.02	2.1	1.8	21.6
	Step 3	176	0.08	0.1	26.6	0.4	75.9	0.25	2.35	<0.1	2.9
	Step 4	12792	350	7.7	254.7	5.0	16071.5	1.01	4.6	33.1	53.5

R1	Step 1	509.2	0.28	6.6	20.9	<0.1	14.3	<0.02	4.9	1.1	0.6
	Step 2	1582.5	0.55	3.4	10.9	<0.1	4345.1	<0.02	2.8	4.7	118.7
	Step 3	935.8	<0.05	0.3	106.1	1.0	191	0.07	2.3	<0.1	0.5
	Step 4	12577.5	55.1	2.0	60.1	2.3	10809.7	0.03	2	86.8	75.2

R2	Step 1	321.5	0.24	0.8	1.1	<0.1	13.2	<0.02	2.5	0.9	0.5
	Step 2	1470.9	1.36	0.9	1.5	0.2	3362.4	<0.02	1.7	7.9	23.9
	Step 3	307.2	<0.05	<0.1	9.5	1.3	46.9	0.14	1.9	<0.1	0.2
	Step 4	11475	61.7	2.8	22.3	6.1	13387.8	0.03	5.7	45.9	79

R3	Step 1	244.8	0.28	1.7	3.3	<0.1	10.1	<0.02	1.9	0.8	61.5
	Step 2	1257.4	1	1.3	3.2	<0.1	3567.8	<0.02	1.3	7.1	38.3
	Step 3	253.3	<0.05	0.2	10.7	0.8	51.4	0.10	1	<0.1	6.8
	Step 4	7846.4	47.5	2.2	25.6	4.0	11631.7	<0.02	2.8	32.6	43.3

R4	Step 1	413.9	0.36	0.2	<0.1	<0.1	48.3	<0.02	1.2	1.2	0.7
	Step 2	1833.6	0.96	0.5	0.4	0.1	2293.2	<0.02	0.9	5.6	38.3
	Step 3	465.6	<0.05	<0.1	2.1	2.2	70.4	0.06	0.9	0.6	0.4
	Step 4	11617.3	55.5	2.4	14.1	4.4	12319.8	0.03	3.4	40.5	86.4

**Table 4:** Varimax rotated for all sediment samples of the Jequetepeque River basin (*n* = 13). Values below 0.3 are not shown.

Metals	Principal component				Communalities
	1	2	3	4	
Zn	0.932				0.981
Mn	0.922	−0.342			0.976
Cr	−0.702		0.520		0.813
Ni	0.541	0.448	0.364	0.465	0.842
As		0.922			0.927
Fe		0.910		0.316	0.979
Cd	0.633	0.648	0.321		0.924
Sn			0.877		0.825
Hg			0.873		0.870
Pb	0.442	0.307	0.655	−0.508	0.976
Al				0.880	0.836
Sb	−0.336		−0.308	0.787	0.832
Cu	0.435	0.502		0.705	0.946
Eigenvalues	3.446	2.990	2.683	2.608	
Cumulative %	26.504	49.506	70.148	90.208	

**Table 5:** Expressions of samples for each cluster in terms of  Varimax rotated factors.

	Factor 1	Factor 2	Factor 3	Factor 4
Cluster 1:				
R2	−0.109	0.070	0.987	−0.524
R3	0.029	0.288	−0.533	−1.469
R4	−1.152	−0.208	0.845	−0.985

Cluster 2:				
Y5	−0.556	2.169	0.484	0.670

Cluster 3:				
Y2	−0.003	−1.388	1.378	1.137

Cluster 4:				
R1	2.368	0.013	0.193	−0.327

Cluster 5:				
Y1	−0.774	−1.106	−1.168	−0.210
Y3	−0.203	0.072	−1.322	0.065
Y4	0.141	0.090	−0.864	1.643
